# Imaging Functional Recovery Following Ischemic Stroke: Clinical and Preclinical fMRI Studies

**DOI:** 10.1111/jon.12668

**Published:** 2019-10-13

**Authors:** Andrew Crofts, Michael E. Kelly, Claire L. Gibson

**Affiliations:** ^1^ Department of Neuroscience, Psychology and Behaviour University of Leicester Leicester UK; ^2^ Preclinical Imaging Facility, Core Biotechnology Services University of Leicester Leicester UK; ^3^ School of Psychology University of Nottingham Nottingham UK

**Keywords:** Stroke, imaging, preclinical, recovery, plasticity

## Abstract

Disability and effectiveness of physical therapy are highly variable following ischemic stroke due to different brain regions being affected. Functional magnetic resonance imaging (fMRI) studies of patients in the months and years following stroke have given some insight into how the brain recovers lost functions. Initially, new pathways are recruited to compensate for the lost region, showing as a brighter blood oxygen‐level‐dependent (BOLD) signal over a larger area during a task than in healthy controls. Subsequently, activity is reduced to baseline levels as pathways become more efficient, mimicking the process of learning typically seen during development. Preclinical models of ischemic stroke aim to enhance understanding of the biology underlying recovery following stroke. However, the pattern of recruitment and focusing seen in humans has not been observed in preclinical fMRI studies that are highly variable methodologically. Resting‐state fMRI studies show more consistency; however, there are still confounding factors to address. Anesthesia and method of stroke induction are the two main sources of variability in preclinical studies; improvements here can reduce variability and increase the intensity and reproducibility of the BOLD response detected by fMRI. Differences in task or stimulus and differences in analysis method also present a source of variability. This review compares clinical and preclinical fMRI studies of recovery following stroke and focuses on how refinement of preclinical models and MRI methods may obtain more representative fMRI data in relation to human studies.

## Introduction

Cerebral ischemic stroke is one of the most common causes of adult death or disability, accounting for 1 in 17 deaths in the United States alone.^1^ Currently, the only approved drug treatment for ischemic stroke is tissue plasminogen activator (tPA), which is only effective for a subset of stroke patients and must be administered within the first few hours to be effective. For the majority of stroke patients, treatment tends to focus on preventing further strokes, for example, through modifiable risk factors, and aiding physical recovery. Functional deficits can be improved to some extent through physical therapy; however, effectiveness varies greatly between patients, and many unknown factors influence the outcome of rehabilitation.^2^ A greater understanding of how functional recovery progresses and what factors promote or impede it would benefit stroke patients.

Preclinical models of stroke have been developed with the aim of improving treatments for, and therefore recovery of, stroke patients. Such models have provided an invaluable insight into the pathological mechanisms activated following ischemic stroke, including the molecular mechanisms of excitotoxicity, the inflammatory response, disruption of the blood brain barrier, and spreading depolarization.^3^ However, these studies have not yet translated into successful clinical treatments. Recovery in rodents is typically measured by a variety of somatosensory tests, ranging from simple tests of sensory function (forepaw reaching, raising paw in response to movement), balance (circling motion) and strength (ability to grip and climb), to more complicated tasks such as the staircase test or the sticker removal test.^4^, ^5^ Although there is a limitation of being unable to measure fine motor control,^5^ Impairment caused by experimental stroke, and degree of functional recovery, can be measured in terms of performance compared to healthy controls (eg, in the sticker test or staircase test), performance compared to prestroke values (ie, baseline), or graded on a set scale (neurological score).^5^ In terms of brain function and connectivity, recovery can be defined as the formation of new connections in surviving neurons that perform the function of the lost neurons.^6^

Functional magnetic resonance imaging (MRI), a noninvasive imaging method, is commonly used in preclinical and clinical studies to detect changes in brain activity. Functional MRI relies on the magnetic properties of oxyhemoglobin and deoxyhemoglobin to detect localized changes in blood flow caused by neuronal activity (the blood oxygen‐level‐dependent [BOLD] response).^7^ When neurons are active, blood flow to the region increases to meet the increased demand for oxygen through neurovascular coupling.^8^ This allows activity in response to a task or stimulus to be localized and quantified, using the BOLD response as a proxy for neuronal activity. As functional MRI (fMRI) does not use ionizing radiation, chemical tracers, or require invasive surgery/implants, it is an ideal method for clinical studies into neurovascular pathologies such as stroke. However, preclinical fMRI has various limitations, including the need for animals to be anesthetized, which impacts upon the utility of fMRI in preclinical studies. Recent advances have allowed the use of awake animals for fMRI scanning,^9^, ^10^ but this requires extensive training of animals, which is not always compatible with disease models and therefore the majority of studies rely on animals being anesthetized prior to scanning. However, many anesthetics, such as isoflurane and halothane, diminish vascular reactivity and dampen the BOLD response.^11^ The commonly used injectable anesthetics urethane and alpha‐chloralose, which do not have this effect, are toxic and carcinogenic, with a high mortality risk after a single dose,^12^ and therefore can only be used in nonrecovery experiments.^13^, ^14^ The use of separate groups for each time point prevents tracking of disease progression, and leads to much higher variability between time points.^15^ To improve the methodology of preclinical fMRI to allow their utility in, for example, preclinical studies of ischemic stroke, it is relevant to consider the key methodological developments that can be gained from clinical stroke studies using fMRI. The ultimate aim is to gain a clear understanding of the processes that may be involved in functional brain recovery following experimental stroke to allow mechanistic investigations that may allow the identification of novel therapeutic targets and ultimately result in patient benefit.

## Functional Imaging of Recovery Following Ischemic Stroke in Humans

Recovery of physical or cognitive function following ischemic stroke has been largely attributed to neuronal reorganization to compensate for lost connections.^6^ Initial studies reported an increase in task‐related activation in the ipsilesional sensorimotor and premotor cortex, the contralesional cerebellum, the bilateral supplementary motor cortex, and the parietal cortex as an indicator of functional recovery.^16^ These increases in activity are thought to represent the disinhibition of existing neuronal pathways,^16^ as other brain regions adapt to allow the patient to relearn the processes required for normal motor function.^17^, ^18^ This adaptation can include bilateral activation, with redundant pathways across the hemispheres becoming active as regions in the undamaged hemisphere replace lost activity in the damaged region.^19^, ^20^ Alternatively, surrounding regions can adapt to bypass damaged pathways. For example, if the corticospinal tract, which connects the motor cortex with motor neurons projecting from the spinal cord, is damaged, alternative pathways via the anterior limb of the spinal cord can be recruited;^21^ however, this adaptation may not result in full restoration of function.

Following ischemic stroke, several studies using either fMRI or positron emission tomography (PET) have described a clear progression of recovery of motor function, at a single time point poststroke, typically 3‐6 months.^22^, ^23^, ^24^, ^25^, ^26^, ^27^ Motor impairment in stroke is usually confined to one side of the body, and changes are referred to as being either contralateral (ie, opposite) or ipsilateral (ie, same) to the side of the ischemic lesion. In terms of comparing motor ability, these studies have displayed an increase in contralesional sensorimotor cortex activation during impaired hand movements, with a similar level of contralesional activation to that seen during unimpaired hand movements. Impaired hand movement also caused near symmetrical activation of the cerebellum^22^ and increased activity in the bilateral parietal cortex, prefrontal and anterior cingulate cortex,^24^ and premotor cortex.^26^ This suggests that a greater degree of voluntary control from these regions compensates for lost autonomic control from the motor cortex as motor function is relearned.^24^ Two of these studies^22^, ^24^ were also notable for including the first analyses of functional connectivity following stroke, and showed that connectivity of motor regions in the cortex and striatum in healthy controls was confined to separate hemispheres, while in stroke patients, this connectivity became bilateral. However, these studies only imaged patients at one time point, and while strength testing showed full recovery in patients, several reported a deficit in their fine motor abilities.^22^ These studies show that changes in activity and regional Cerebral Blood Flow (rCBF) occur across both hemispheres following stroke, yet longitudinal studies are required to monitor progression in recovery.^22^, ^23^, ^24^

Longitudinal studies tend to begin in the first few weeks poststroke and continue for several months to a year poststroke. Initially, these studies built on the work of Nelles et al^27^ by imaging patients at the acute stage (<20 days post stroke) while still suffering from hemiparesis, with a further scan occurring, for example, 3 weeks later,^28^ or up to 6 months poststroke.^21^ Between 2 and 5 weeks poststroke, the main change in activation patterns was a decrease in CBF response and size of activated area in ipsilesional regions, correlating with improvement in limb function.^28^ Thus, although previous studies using a single time point poststroke demonstrated that contralesional activity following stroke contributed to compensation for lost connections in the impaired hemisphere,^22^, ^24^, ^25^, ^26^ longitudinal studies suggest that this contralesional activity was only the first stage of recovery.^29^ In this first stage, a reorganized motor network that formed within 24 hours of the ischemic insult was described, allowing the damaged pathways to be bypassed through recruitment of contralesional pathways^30^ or alternative pathways in the brainstem and spinal cord.^21^ Reorganization of the impaired hemisphere and restoration of activity around the lesion was shown to be the next important stage of recovery, in which contralesional activity supports these new, less efficient connections while processes are relearned.^2^, ^17^, ^29^ The most complete recovery, in a neurological and behavioral sense, would be a return to mostly unilateral activity in the motor cortex, with the center of activation shifting to accommodate the damaged tissue, and the BOLD signal intensity returning to the same level as the undamaged hemisphere.

Based on information gained from both short‐term and long‐term assessment of recovery following stroke, it may be possible to define two distinct phases of recovery in brain activity—recruitment and focusing.^28^ The recruitment phase increases the population of available neurons to compensate for lost connections, particularly supplementary cortical regions and contralesional regions. For example, Ward et al^19^ showed in stroke patients that activity in sensorimotor regions was negatively correlated with outcome in subcortical stroke, and thus if the high activity observed in the recruitment phase persisted, motor ability did not recover to prestroke levels. In patients displaying better task performance, no significant positive correlation between task outcome and task‐related sensorimotor activity was found, showing that focusing of activity was the main indicator of good recovery in the group. A subsequent study by the same group^18^ focused on a second, smaller group of patients over a longer time period (6 months), and found the same distribution of hyperactivation, focusing, and reorganization of motor activity.

The connections utilized in the recruitment phase are thought to be present but inhibited in healthy brains, which are disinhibited following stroke.^31^ Models of learning in healthy subjects suggest a similar process in which a large region is recruited, followed by focusing as pathways become reinforced.^32^, ^33^, ^34^ Thus, the process of learning leaves redundant pathways in supplementary regions that can be recruited in the event of injury, allowing for the brain to be highly adaptive in the event of, for example, ischemic stroke. In stroke patients with motor deficits, this manifests as activity in the inferior parietal cortex,^16^ a region known to be important in early stages of motor learning and the ability to visualize a task,^35^ suggesting that improved sensory processing may act to compensate for motor deficits.

In the focusing phase, as processes are relearned, activity is reduced toward baseline levels as connections become more efficient, and activation in contralesional or supplementary regions is reduced or eliminated.^19^, ^20^, ^29^ Reduction in activity in the cerebellum as recovery progresses^16^ also correlates with changes observed in healthy subjects learning a new motor task.^36^ However, lesion size and location affect the time course of these phases, with some patients never progressing to the focusing phase.^17^

More recent studies using resting‐state (RS) fMRI have identified changes in functional connectivity that correlate with recovery. Interhemispheric connectivity greatly reduces in the acute stage following stroke, even in regions outside the lesioned area.^37^, ^38^ This is thought to initially be due to the undamaged hemisphere influencing the damaged hemisphere, as undamaged regions compensate for lost tissue, with connectivity becoming more balanced with recovery,^18^, ^39^ fitting with the model of increased recruitment followed by focusing derived from task‐based studies.^29^

RS‐fMRI has certain advantages over task‐based fMRI. Different studies may measure task performance differently, for example, in hand grip tasks. It has been suggested that how the maximum contractile strength is calculated, whether using the first task as a set maximum or calculating a new maximum with each session, which affects the target grip strength so can affect the measured outcome.^18^ Hand movement tasks, for example, flexion‐extension, vary greatly in complexity, and can be active, in which the patient performs the movement themselves, or passive, in which the researcher moves the limb by a set amount.^14^ A third task type, fine motor control, for example, using finger‐thumb opposition,^19^ can also vary in complexity, which can influence the size of the active region and lateralization of activity.^38^ RS‐fMRI removes this factor, so greater focus on RS‐fMRI may facilitate better comparison between studies and further understanding of recovery mechanisms that would not be discovered through task‐based fMRI.

Through the use of fMRI, the progression of brain reorganization and functional recovery following ischemic stroke has been well documented in humans. However, fMRI studies in animal models, which are an important step in the translational pipeline for identifying novel disease targets, are in their infancy and have a number of methodological limitations to overcome. Rodent studies of ischemic stroke use a variety of methods to analyze outcome including behavioral measures, electrophysiology, structural imaging, and more recently, functional imaging. Such rodent studies should ideally identify comparable alterations in brain function and connectivity between rodent stroke models and human stroke patients.

## Functional Imaging of Recovery Following Ischemic Stroke in Rodents

Very few preclinical stroke recovery studies have used fMRI as an outcome measure (summarized in Table 1),^40^, ^41^, ^42^, ^43^, ^44^, ^45^, ^46^, ^47^, ^48^, ^49^, ^50^ which may be due to limitations of equipment availability, financial cost, technical expertise, and uncertainty regarding the fMRI protocol with regard to appropriate anesthesia. Human stroke patients are typically awake during fMRI scanning but it is common practice for animals to be anesthetized. Thus, in rodents, fMRI typically requires the use of an appropriate anesthetic protocol that allows for detection of a hemodynamic response and for animals to recover so that they may be scanned at sequential time points and/or used for additional outcome measures, such as behavior. Because of this, preclinical fMRI studies of poststroke recovery have tended to use different groups of animals for each time point,^40^, ^41^, ^44^ introducing an additional source of variability and limiting the ability to monitor the progression of pathology and/or recovery in brain function over time.

**Table 1:** Summary of Methods and Outcome Measures of Recent Preclinical fMRI Studies of Experimental Stroke in Rats’ Study

	Rat Strain/Sex/Age	Longitudinal?	Anesthetic	Stroke Model	Outcome Measures
Dijkhuizen et al^40^	Sprague‐Dawley, male, adult	No	Alpha‐chloralose	Permanent MCAO	Cerebral blood volume fMRI (3/14 days post‐MCAO), behavior, lesion volume.
Abo et al^45^	Sprague‐Dawley, male, young adult	No	.5% Isoflurane, muscle relaxant	Photochemical	Cerebral blood volume fMRI (3 weeks poststroke, behavior).
Sauter et al^43^	Fischer, male, not specified	No	2% Isoflurane/Nitrous oxide	Permanent MCAO	CBF fMRI, lesion volume (1/2/5/12 days post‐MCAO), apparent diffusion coefficient (ADC). Placebo vs neuroprotection with isradipine.
DIjkhuizen et al^41^	Sprague‐Dawley, male, adult	No	Alpha‐chloralose	2 hour MCAO	CBV fMRI (1/3/14 days post‐MCAO), neurological score, lesion volume, ADC
Markus et al^46^	Not specified, not specified, 25 months	No	Not specified	Permanent MCAO	CBV fMRI (8 weeks post‐MCAO), behavior, effect of novel therapeutic
Sicard et al^100^	Sprague‐Dawley, male, not specified	Yes	1% isoflurane	20 minute MCAO	BOLD fMRI, behavior, lesion vol, ADC (0 minutes/30 minutes/180 minutes/1 day/7 days/21 days post‐MCAO)
Kim et al^47^	Sprague‐Dawley, male, adult	No	Alpha‐chloralose	2 hour MCAO	BOLD and CBV fMRI (2 weeks post‐MCAO), lesion volume
Kim et al^48^	Sprague‐Dawley, male, not specified	No	Alpha‐chloralose	90 minute MCAO	BOLD and CBV fMRI (2 weeks post‐MCAO), lesion volume, effect of IV albumin.
Weber et al^42^	Wistar, male, not specified	Yes	Medetomidine	1 hour MCAO	BOLD fMRI, electrophysiology (2/7/14/28/49 days post‐MCAO)
Van Meer et al^43^	Sprague‐Dawley, male, not specified	No	1% isoflurane	90 minute MCAO	RS‐fMRI, manganese‐enhanced MRI
Van Meer et al^49^	Wistar, male, not specified	Yes	1.8% isoflurane	90 minute MCAO	RS‐fMRI (3, 7, 21, and 70 days post‐MCAO), lesion volume, manganese‐enhanced MRI, behavior
Van Meer et al^60^	Sprague‐Dawley, male, young adult	Yes	1% isoflurane	90 minute MCAO	RS‐fMRI (3, 7, 21, 49, 70 says post‐MCAO), lesion volume, EEG, behavior
Shih et al^98^	Sprague‐Dawley, male, adult	Yes	1% isoflurane	20 or 45 minute MCAO	CBV fMRI, electrophysiology (0/7/21 days post‐MCAO)
Lake et al^55^	Sprague‐Dawley, male, adult	Yes	Propofol	Endothelin‐1 injection	CBF fMRI with hypercapnic stimulus, electrophysiology with forepaw stimulus (7 and 21 days post‐MCAO)
Lake et al^83^	Sprague‐Dawley, male, adult	Yes	Propofol	Endothelin‐1 injection	CBF fMRI with hypercapnic stimulus (7 and 21 days post‐MCAO), effect of COX‐1 inhibitor
Shim et al^50^	Sprague‐Dawley, not specified, adult	No	Alpha‐chloralose	2 hour MCAO	BOLD fMRI, RS‐fMRI, Diffusion‐weighted MRI, lesion volume (6 months post‐MCAO)

Initial functional MRI studies performed in rats, using nonrecovery anesthesia and invasive monitoring protocols, showed low levels of contralesional activity in response to forepaw stimulation at both 3 days and 14 days poststroke, which was not seen in sham animals, and an initial reduction in ipsilesional activity at 3 days, followed by a slight increase at 14 days poststroke.^40^ This initial increase in contralesional activity, followed by a decrease as ipsilesional activity is restored, is similar to the changes seen in human stroke patients.^20^ However, in human studies, contralesional activity is higher at the early‐stage poststroke, and ipsilesional hyperactivity is exhibited as recovery progresses,^16^ rather than the low levels reported in rodents. Similar findings were reported in a subsequent study by the same group,^41^ although both studies report high intersubject variability, which was also seen when animals were exposed, under isoflurane, to nonrecovery scans poststroke.^44^ Studies using a single time point also detected contralesional activity, using Cerebral Blood Volume (CBV)‐weighted or contrast‐enhanced MRI sequences.^45^, ^46^, ^47^, ^48^ These methods, when applied to studies of novel therapeutics with behavioral outcome measures, showed benefits of modulating pathways involved with plasticity in the thalamus^46^ and the contralesional cortex^48^ on outcome in the chronic phase. Single time‐point studies such as this are beneficial when performed in conjunction with longitudinal behavioral studies, in understanding how differences in activation patters correlate with long‐term behavioral outcome. However, their ability to infer the mechanisms behind functional recovery is limited.

Advances in poststroke MRI in rodents have been assisted with the development of suitable anesthetic protocols for longitudinal functional studies, for example, using a subcutaneous infusion of medetomidine.^51^ A longitudinal study using this method showed three different patterns of activity following induction of experimental stroke via middle cerebral artery occlusion (MCAO).^42^ All animals underwent the same MCAO procedure; however, one group of animals showed no loss of BOLD response to forepaw stimulation and one group showed a complete loss of activity in the forepaw region of the ipsilesional somatosensory cortex (S1FL). These results suggested that there is high variability in the effects of MCAO on the injured hemisphere. Across all groups, somatosensory evoked potential (SSEP) signals were consistent with the observed BOLD activity, suggesting preservation of neurovascular coupling. In contrast to previous studies,^16^, ^40^, ^41^ there was no contralesional somatosensory cortex activity in response to impaired forepaw stimulation. Lesion volume over time correlated with changes in BOLD and SSEP signals, with small subcortical lesions causing no significant deficit, and loss and recovery of the BOLD response correlating with size and recovery of the lesion.^42^ Thus, this tended to suggest three different severities of stroke as a consequence of the same MCAO procedure—minimal damage, severe damage with recovery, and severe damage with no recovery.^42^ The group exhibiting complete loss of activity in the ipsilesional cortex contrasts with human studies, as impaired forepaw stimulation did not evoke contralesional activity, and there was no period of ipsilesional hyperactivity as observed in humans.^16^, ^19^, ^20^ The variability in lesion volume and outcome, despite all rats undergoing the same MCAO surgery, shows an inherent problem associated with such experimental stroke models. Although attempts are being made to try and reduce the variability in outcome measures, such as lesion volume, associated with the MCAO model of experimental stroke,^52^ this does emphasize the importance of performing longitudinal fMRI studies to account for within‐group variability caused by the MCAO method, and minimizing sources of variability in the imaging protocol.

A recent study compared fMRI with local field potential (LFP) responses, using continuous arterial spin labeling MRI to measure the CBF response to hypercapnia under propofol anesthesia.^53^ While the CBF and LFP responses in this case were not directly compared, as forepaw stimulation was performed for LFP experiments while CBF responses were induced with hypercapnia, the two modalities did show changes in poststroke activity resembling those observed in humans.^29^, ^54^ Resting perfusion in the ipsilesional hemisphere showed an initial increase, and returned to baseline at 21 days poststroke.^53^ Reactivity to hypercapnia in the ipsilesional hemisphere was raised at both time points. This was attributed to injury induced angiogenesis, triggered following ischemia,^55^, ^56^ leading to a higher vascular density at 7 days, with excess vessels pruned back in the following weeks. This supports fMRI data in humans showing increased neuronal recruitment followed by focusing of activity.^29^ As new pathways are recruited, additional blood flow is required to support the increased activity, and as these pathways become more efficient, less blood supply is needed.^57^ LFP data showed altered organization in the ipsilesional somatosensory cortex compared to the contralesional cortex. In the undamaged hemisphere, response amplitude to stimulation decreased with distance from Bregma, correlating with the responses mapped in the cortex of healthy rats.^58^ Following stroke, this correlation is lost, and as recovery progresses, the reverse is true, with peak responses observed at the furthest distance from Bregma.^53^ This supports the previous data shown in humans of reorganization, where the peak activity detected is shifted compared to healthy controls as recovery progresses.^20^

In recent years, RS‐fMRI has become a more common outcome measure in both clinical and preclinical research. The ability to examine connectivity between brain regions is beneficial to the understanding of recovery following stroke, as supplementary regions compensate for ischemic tissue and alternative pathways are formed. Sources of variability related to an external stimulus, such as sensitization of the paw from electrode placement, artifacts from the electrical current, or habituation in the brain to the stimulus paradigm, are also avoided. As with stimulus‐based fMRI, there has been a mix of single time point and longitudinal RS‐fMRI studies performed.^43^, ^49^, ^50^, ^59^ Results of RS‐fMRI studies show more consistency than stimulus‐based fMRI, and the progression of recovery in rodent RS‐fMRI studies shows more features of the progression seen in humans. For example, increase in recruitment in early stages followed by focusing of activity as recovery progresses can be seen in one study of functional connectivity, in which connectivity between the somatosensory and motor cortices in the ipsilesional hemisphere is increased above baseline at 3 weeks, before returning to baseline at 10 weeks.^43^ Other studies show a correlation between improved interhemispheric functional connectivity in the chronic phase and improved functional connectivity,^50^, ^51^, ^59^ and this is supported by a study treating experimental stroke with mesenchymal stem cells (MSCs), in which MSC‐treated groups show improved interhemispheric connectivity and behavioral outcome.^60^ While animals are grouped by stroke severity, it is important to note that lesion volume or localization (ie, subcortical or subcortical + cortical) have an impact on outcome,^49^, ^59^ with animals exhibiting lesions covering both cortical and subcortical regions not showing the recruitment and focusing pattern.^43^ This further supports the need for stroke studies to group animals according to stroke severity, to account for variability and allow therapeutic studies to investigate different treatments to fit the severity of the lesion.

While rodent fMRI studies have begun to show important insights into recovery of brain function following experimental stroke, the limited number of studies and the methodological variations between studies make comparisons difficult. However, the additional use of more invasive techniques such as electrophysiology, which typically cannot be used in humans, does allow parameters to be measured in rats that cannot be measured in humans. For example, this may allow insights into which aspects of recovery are neuronal and which aspects are vascular.^53^ If preclinical studies using disease models wish to focus on measuring recovery, then improvements in preclinical imaging methodology may allow more consistent measures of functional recovery that correlate with those seen in human imaging studies.

## Methodological Considerations Relevant to Preclinical Stroke Imaging

Several factors are important to consider in relation to the sources of inconsistencies between preclinical stroke imaging studies and trying to improve correlation with clinical studies. The first is whether a study uses several groups of animals at individual time points or conducts longitudinal analyses. In recent years, longitudinal studies have begun to emerge, thus reducing variation between time points, allowing progression in individual animals to be tracked, and reducing the overall number of animals needed.^42^, ^43^ Although appearing advantageous, there are methodological limitations to consider for longitudinal studies as they require multiple anesthetic use that may cause safety and ethical concerns. In addition, an anesthetic protocol, or reliable training method if using awake animals, needs to result in minimum impairment of the BOLD signal so that changes detected are a true representation of neurological activity and neurovascular coupling. In addition, the method of stroke induction is important, in order to ensure sufficient damage to impair function, while trying to minimize the impact in terms of welfare. Ethical concerns may arise when considering the impact of repetitive scanning on the cumulative severity an individual animal is exposed to over their lifetime and ethical guidance is likely to vary between institutions and countries. Recently published guidelines suggest methods in which variability can be reduced and welfare improved.^61^ Following these guidelines, in conjunction with the Animal Research: Reporting of In Vivo Experiments (ARRIVE) guidelines on reporting in animal experiments,^62^ it would be beneficial for animal welfare, experimental outcomes and analysis, and reproducibility. The ARRIVE guidelines aim to standardize methods of reporting to cover aspects of animal work not previously reported. This includes a more detailed description of study design, and the reasons behind choices such as surgical method or anesthetic agent, which would be beneficial to attempts to standardize methods across different groups. Reporting of housing and husbandry conditions, welfare concerns that arose and how they were dealt with, additional details of procedures such as time and location, and animal health measures such as weight and diet prior to procedures may also aid in identifying sources of variability. The IMPROVE guidelines suggest a number of improvements to housing procedures specifically for the welfare of pre‐ and poststroke animals, and implementing these changes may mitigate sources of variability related to animal health prestroke,^61^ and reporting these methods can lead to identification of new environmental risk factors to study.

Another issue that relates both to animal welfare and scientific outcome is in the timing of experimental work, and in balancing a minimized impact on animal welfare with a robust model of stroke progression. Factors to consider include the age at which experimental stroke is performed, housing procedures following stroke, the timing of any intervention, or the timing of behavioral and MRI experiments.^61^ In humans, age of onset is a major determinant of recovery following stroke, and stroke is most common in old age.^63^ In rodent studies, however, rats are generally given experimental stroke at a young age. This also applies to models of other age‐related diseases, most likely due to the time, expense, and welfare risks of aging animals prior to study. Improvements to animal housing and welfare for stroke animals also have applications in aging animals, and through applying these to ensure that animals remain healthy as they are, older animals can be used to understand the effect of age in preclinical stroke.^61^ The length of the study poststroke, and how enrichment, imaging experiments, behavioral testing, and other interventions are timed, is also an important factor. The time course of recovery is heterogeneous, and in humans’ recovery, it is often measured by the return of abilities such as fine motor control. Human studies then tend to group patients according to their level of this functional recovery, through performance in a task, to account for the variable time course.^16^, ^17^, ^18^, ^19^ Longitudinal rodent studies, on the other hand, tend to measure progression in fMRI and behavioral outcome measures over time, showing a group average with high variability.^42^, ^43^, ^44^, ^45^, ^46^, ^47^, ^48^, ^49^, ^50^ Grouping animals by behavior, or lesion volume, and then analyzing fMRI results over time would go a long way toward accounting for the high variability observed, and in understanding the complex interplay between structural damage, neurovascular function, and behavioral outcome.^42^ This also allows for better timing of interventions, or enriching their environment poststroke, to be appropriate for the animals’ state of recovery.

It has also been shown that improved reperfusion following MCAO can reduce variability in lesion size, as well as reducing mortality and being beneficial for animal welfare.^52^ Other aspects of MCAO to consider include understanding the differences between animals that may influence the severity of the damage from MCAO. In particular, variations in the degree of collateral perfusion surrounding the circle of Willis are thought to be a major factor determining the size of an ischemic lesion,^64^ and research into this variation in rodents may aid in predicting this variability before surgery, and grouping animals accordingly. As increasing occlusion time does increase the chance of a larger infarct,^42^ studies into the range of lesion volumes caused by different occlusion times in transient MCAO can also account for variability between studies. Modifications such as these to MCAO studies to reduce variability in, for example, lesion size may make comparisons across studies easier and allow the effect of lesion volume on recovery progression to be studied further.

Another method suitable for studies of recovery is the photothrombotic model. This model uses application of a photosensitive dye into the cerebral vasculature, which is activated by a laser to triggering platelet aggregation. This gives a controlled infarct size with low mortality, does not require surgery, and allows control over the exact location of the infarct. Reperfusion can be triggered by a second laser.^65^ This model does not produce a penumbra; however, there are variants that produce a similar effect.^66^ The control of size and location of the infarct allows modeling of ischemia in less common locations than the MCA. This model may be useful in studies of less common forms of cerebral stroke and studies of plasticity in the chronic phase, with fewer welfare risks than surgical methods, at the cost of modeling the penumbra. The advantages and disadvantages of the various approaches used to model ischemic stroke in rodents have been further reviewed elsewhere including methods that do not allow reperfusion and therefore are more suitable for neuroprotection studies rather than studies of recovery.^67^

Several studies have focused on developing an appropriate anesthetic protocol for fMRI. Urethane and alpha‐chloralose are the two most commonly used injectable anesthetics for fMRI, and while they both allow for robust BOLD responses, their carcinogenic effects prevent their use for longitudinal experiments.^14^ For longitudinal fMRI, some studies use a continuous infusion of medetomidine. BOLD responses to forepaw stimulation, with two sessions under medetomidine or alpha‐choralose, showed clear, reproducible changes with no significant difference between signal intensity under medetomidine or alpha‐chloralose.^51^ However, medetomidine does introduce some confounding factors, as it produces a variable depth of anesthesia.^68^ It has been shown that long‐term anesthesia with medetomidine causes an increase in heart rate over time, and an increase in dose rate is required to maintain anesthesia.^68^ Medetomidine can also cause hypoxia, and when the method was first described, oxygen had to be increased to 30% part way through scans.^51^ The use of a low dose combined with another sedative, such as ketamine, has been recommended instead of medetomidine alone in scientific and veterinary settings.^14^, ^69^ Additionally, in functional connectivity studies, animals show impaired connectivity compared to the awake state or other anesthetics, with all regions showing a reduction in correlation coefficients, and the thalamo‐cortical pathway showing complete loss of activity.^70^

In terms of inhalational anesthetics, some recent fMRI studies report using low doses of isoflurane anesthesia.^43^ However, even low doses can affect the BOLD signal, which compromises the accuracy of results, particularly in longitudinal studies, as a reduction in signal amplifies the effects of physiological noise and interanimal variation.^11^ Long‐term changes may also be lost, for example, where hyperactivation may be observed in the awake animal, isoflurane will reduce the maximum signal change that can be observed, masking this effect.^71^ The effect of isoflurane on BOLD signal is quite complex with dose‐dependent effects on several different factors contributing to the signal, for example, isoflurane reduces local cerebral glucose utilization (LGCU) by 40‐60%, while increasing resting CBF by 25‐60% measured using radiolabelling methods.^72^ However, BOLD signal intensity does not change linearly with dose, instead signal intensity peaks between 2% and 2.5% isoflurane and drops as the dose is either reduced to 1.5% or increased to 3%,^73^ which is thought to reflect the different effects of isoflurane on arteries and microvasculature. BOLD responses are the same in the arteries and microvasculature at 1.5% and 3% isoflurane, but at 2‐2.5%, the arterial BOLD signal is larger.^73^ Even .5% isoflurane showed a significant drop in evoked BOLD signal, with one study comparing awake animals with animals under .5% isoflurane showing a 50% decrease in BOLD signal under .5% isoflurane, while LFP and multiunit activity signals only decreased by 10%.^71^ Halothane also has similar effects on stimulus‐based BOLD signal, and is also considered unsuitable for use in fMRI studies.^74^ There is evidence to suggest that isoflurane may be suitable for RS‐fMRI studies, depending on the dose rate and regions being investigated. Cortical functional connectivity is well preserved under isoflurane.^70^ However, fluctuations in subcortical regions are impaired in a dose‐dependent manner, requiring less than 1% isoflurane for any subcortical or thalamocortical networks to be identified.^75^, ^76^

Propofol, while less commonly used than medetomidine, isoflurane, or urethane, has been suggested as a good candidate for preclinical fMRI studies.^70^, ^77^, ^78^, ^79^ Studies of hypercapnia show that cerebral autoregulation and cerebrovascular reactivity are maintained under propofol while they are lost under isoflurane.^80^, ^81^ A study comparing isoflurane, medetomidine, propofol, and urethane showed signal intensity under propofol to be three times greater than under medetomidine, and a hemodynamic response function (HRF) closer to that of the awake state.^82^ Studies using arterial spin labeling have also shown that propofol gives a clear CBF response, and comparison between studies by the same group using propofol and medetomidine shows a greater baseline CBF and stimulus‐induced CBF response under propofol than medetomidine.^78^, ^79^ As functional connectivity responses, cerebral autoregulation, cerebrovascular reactivity, and the HRF under propofol are shown to be close to that of the awake state, propofol appears to be an ideal choice of anesthetic protocol for fMRI. However, to date, use of propofol in disease models such as ischemic stroke is limited to a few studies observing changes in global CBF in response to hypercapnia.^53^, ^83^

While for practical reasons, anesthetizing animals for MRI studies has been the norm, protocols have been developed for scanning awake animals.^9^, ^71^ The benefit of this is clear, rendering animals unconscious affects brain and vascular activity, so functional responses in an anesthetized animal are different from those in an awake animal. Similarly, if comparing human and animal studies, using anesthesia introduces a new confounding factor, as humans are usually awake when undergoing scanning. When studies have compared awake and anesthetized animals, regardless of anesthetic used, a greater signal change and activated area were found in the awake condition, with propofol giving the next largest BOLD signal change.^71^, ^77^, ^84^ Different anesthetic protocols also alter network activation,^70^ and can have a variety of adverse effects on blood pressure, breathing, oxygen saturation, and temperature, depending on choice of agent and dose.^14^ Anaesthetizing animals does mean that movement is minimized, thus preventing loss of image quality and signal artifacts. It also prevents stress from the sound of the MRI scanner, reflexive movement in response to stimulation, and discomfort from attachment of electrodes or physiological monitoring equipment. However, there are several problems in implementing an awake imaging protocol in stroke studies. Awake rodents have not, to date, been reported in functional imaging studies following induction of stroke, or in other age‐related neurological disease models. Thus, it is unclear if, or how stroke may affect acclimatization to the scanner and the extensive pretraining required. Training of rats for awake imaging is time‐consuming, with studies performing training sessions daily for at least 2 weeks.^9^, ^10^ Stroke has damaging effects on memory, spatial awareness, motor control, and stress levels, all of which will have a detrimental effect on the habituation state of the animals.^85^ Acclimatizing the animals again poststroke would be impossible due to the animals’ welfare and time constraints. Thus, scanning of awake rodents in stroke studies is not possible at the current time from a welfare perspective and a practical perspective. Rats are known to be more sensitive to stress after stroke,^76^ which may affect the restraint method, and motor deficits may affect the rats’ ability to position themselves appropriately during the restraint period.

While functional imaging does provide insight into changes in activity and connectivity following stroke, other methods are required to investigate the underlying mechanisms. Signaling cascades triggered by spreading depolarization, for example, severely disrupt neurovascular coupling and oxygen metabolism, as well as influencing plasticity and neuronal activity.^86^ These factors all influence the BOLD fMRI signal, but studying these signaling mechanisms requires invasive or ex vivo techniques. While CBV imaging can give an indication of vascular density, studies into angiogenesis in stroke recovery also use molecular or ex vivo techniques.^87^, ^88^, ^89^ Additionally, while recruitment of unused pathways is inferred from the changes in the BOLD signal with recovery, the mechanisms behind disinhibition and inhibition of pathways cannot be measured this way, and so understanding the processes influencing the transition to recruitment then focusing phase would require more invasive methods. However, in recent years, there has been an increase in research combining MRI with other approaches, such as optogenetic MRI,^90^ MRI/PET,^91^ or combining MRI with two‐photon microscopy,^92^ optical image spectroscopy,^93^ or calcium imaging,^94^ which will allow preclinical imaging studies to look further into these signaling and metabolic changes. Applying knowledge of signaling mechanisms behind stroke‐related neurovascular changes to optogenetics would have value in understanding the influence of neurovascular uncoupling on the BOLD signal in initial stages poststroke and pathways influencing recovery in later stages. Concurrent MRI and two‐photon or calcium imaging would give further insight into the balance between angiogenesis and neuronal recruitment alluded to in MRI/electrophysiology studies.^53^ However, these methods either require genetically modified animals or additional surgery, limiting their use in translational research. MRI methods, such as angiography or oxygen 17 imaging, or more novel methods such as functional diffusion‐weighted MRI, chemical exchange saturation transfer (CEST),^95^, ^96^, ^97^ are noninvasive, but require more development to improve resolution or to be feasible in a clinical scanner before they can be applied to translational research.

## Conclusions and Future Directions

Functional recovery in stroke patients is reliant on mechanisms of synaptic plasticity associated with learning. These mechanisms allow new pathways to be recruited and reinforced that circumvent damaged tissue, and thus, restoring the lost functions to a degree. Clinical studies of stroke patients with motor deficits have observed a pattern of recruitment and focusing that determines the extent of recovery of lost functions. Unused synapses are disinhibited around the lesion and in the contralesional hemisphere, as the patients begins to use the impaired limb again. Large regions of activity are seen on fMRI scans during motor tasks, mimicking the process of recruitment seen in motor learning in healthy subjects. As learning progresses, certain pathways are reinforced and unnecessary pathways are inhibited, and this focusing of activity represents the second phase of recovery. Performance on motor tasks following stroke is correlated with which phase of recover a patient displays on fMRI scans. Patients showing poorer motor performance are in the recruitment phase, and some patients do not progress past this phase.^29^ As motor performance returns toward prestroke levels, fMRI scans display more focused activity, shifted to accommodate the area of lost tissue.^16^ While these changes can be observed in clinical studies, to understand the mechanisms behind this activity and determine what factors influence progression of recovery, a preclinical model that displays these features is required.

While, in principle, the noninvasive nature of fMRI should be beneficial in animal models of disease, such as ischemic stroke, but due largely to the practical challenges of preclinical functional imaging, there are currently only a small number of longitudinal studies of stroke. The findings of these studies vary greatly, due to differences in anesthetic protocol and/or method of experimental stroke used.^42^, ^43^, ^53^, ^98^ In addition, none of these studies replicate the recruitment and focusing pattern of recovery seen in human stroke patients. The requirement for, and reliance on, anesthesia in preclinical studies probably accounts for this lack of reproducibility between preclinical and human studies. Increasing prevalence of RS‐fMRI in preclinical studies is a positive step, providing insights into effects of stroke on the brain as a whole in addition to the effects on the area surrounding the lesion. The lack of an external stimulus also removes a source of variability. Greater focus on RS‐fMRI studies would also remove a major difference in methodology between preclinical and clinical studies, in that rodents cannot perform motor tasks during fMRI as humans can, and so rodent studies must use a sensory stimulus. However, the complicated interactions between different anesthetics and functional brain networks are only recently becoming clear. Recent studies have begun to identify signaling mechanisms behind RS fluctuations,^99^ and understanding of these pathways may support choice of one anesthetic over another depending on their interactions. Understanding and minimizing these factors that disrupt functional connectivity needs work to aid in improving preclinical RS‐fMRI studies.

Improvements to current methodology for fMRI studies could address some of the issues identified here and improve the ability of preclinical models of ischemic stroke to demonstrate the same changes in brain function and connectivity seen in human stroke studies. For example, the anesthetic agent propofol, which is common in veterinary medicine and has a minimal effect on HRF and the BOLD response, has the potential to reduce variability in longitudinal fMRI studies, and may allow for the pattern of recruitment and focusing of activity to be detected in rodents. Recent studies also suggest a lesser disruption of functional connectivity under propofol than some other anesthetics; however, further study into the interactions of anesthetics with functional networks would be beneficial. Ideally, animals should be scanned in the awake state, to more closely replicate the methods used in human studies; however, concerns over distress caused to the animal, or whether stroke animals can be acclimatized to conditions in the scanner may prevent this. Thus, it is timely to consider modifications in the methods used for anesthetized animals for fMRI scanning that may improve the translational relevance of preclinical stroke models.
